# Does foreign direct investment affect environmental degradation: Evidence from largest carbon intense countries

**DOI:** 10.1371/journal.pone.0314232

**Published:** 2024-11-21

**Authors:** Ozlem Kutlu Furtuna, Selin Atis

**Affiliations:** 1 Business Administration Department, Center for Finance Governance and Sustainability (CFGS), Yıldız Technical University, Istanbul, Türkiye; 2 Social Sciences Institute, Yıldız Technical University, Istanbul, Türkiye; Harran University: Harran Universitesi, TÜRKIYE

## Abstract

Foreign Direct Investment (FDI) and environmental degradation are some of the most controversial debates, especially in the context of global warming and climate change. This study aims to shed light on the impact of FDI on environmental degradation in the countries with the highest carbon dioxide (CO_2_) emissions, taking into account 513 country-years between the years 1996 and 2022. CO_2_ and the ecological footprint were used as indicators of environmental degradation. The possible non-linear linkage between FDI and environmental degradation has also been analyzed. Gross domestic product (GDP) growth and inflation rate were used as control variables. The results of the panel data analysis show a U-shaped relationship between FDI and carbon emissions which means carbon emissions decrease to a certain level with increasing FDI investment and after this level, increasing FDI increases the environmental degradation in terms of carbon emissions. Moreover, FDI and the non-linear form of FDI have no significant influence on ecological footprint. This study also highlights the importance of international agreements and frameworks, such as the Sustainable Development Goals and the Paris Agreement, in guiding nations towards a more sustainable future. These empirical results are vital for regulators, emphasizing the need for a holistic and multidimensional approach to both economic prosperity and environmental protection.

## Introduction

The decline in environmental quality around the world has made the impact of environmental degradation a controversial issue. Foreign Direct Investment (FDI) plays a crucial role in boosting the growth and development performance of today’s developing countries. Technology transfer and innovation activities have a significant impact, especially for countries with savings gaps, leading to increased competition. The main argument, however, is that these countries are exposed to pollution because industrialized countries are relocating their polluting sectors to developing or underdeveloped countries. Polluting industries are fleeing from countries with strict policies to countries with flexible environmental policies [1]. However, the relocation of carbon-intensive industries from industrialized to developing countries is not only due to weak environmental policies in the host countries. At the same time, cheap labor, access to raw materials, energy consumption, and abundant natural resources in the host countries are decisive factors for industrialized countries when choosing a country to invest in. The comparative advantage in factors of production encourages foreign investors to invest in developing countries [2]. This fact draws attention to the importance of studies on the impact of foreign investment on environmental pollution.

The serious change in the global risk profile has recently had an impact on the world economy. Countries are facing ever greater challenges in connection with the revelations about climate change and economic problems. According to the World Economic Forum’s (WEF) Global Risks Report 2024, among the 10 risks the world will face in 10 years, the top three risks are related to climate change, such as extreme weather events, critical changes to earth systems, and loss of biodiversity [3]. The Intergovernmental Panel on Climate Change (IPCC) argues that climate change is causing widespread loss reducing economic growth and destroying nature. The Paris Climate Agreement has set itself the *goal of reducing carbon dioxide emissions to zero in the second half of the century and limiting the global temperature rise to 1*.*5°C above pre-industrial levels* [4]. Greenhouse gas emissions are one of the most important causes of climate change and global warming [5].

The pollution haven and pollution halo hypotheses are the two main theories for explaining the influence of FDI on environmental sustainability. The pollution haven theory describes how, due to strict environmental laws in the developed countries, the polluting sectors in these countries are relocated to the developing countries through FDI and these developing countries become pollution havens for the developed countries. The pollution haven theory states that strict legal regulations on environmental standards in industrialized countries increase the production costs of companies in these countries. The construction of plants that cause pollution in developing countries through FDI leads to the formation of pollution havens [6]. In other words, pollution occurs in developed countries where strict environmental regulations exist. ​The relocation of industries to developing countries, where environmental policies are inadequate and lax, creates ’pollution shelters’. As a result, environmental problems are increasing in developing countries where environmental awareness is low [7].

Studies investigating the impact of CO_2_ emissions on economic growth are in the majority. This paper aims to draw particular attention to the countries that emit the most carbon, as environmental degradation is more pronounced with an increase in CO_2_ consumption. However, the number of studies that have examined the relationship between FDI and environmental degradation in terms of carbon emissions and ecological footprint from the perspective of Sustainable Development Goal (SDG) 17 priorities, enhancing the global partnership for sustainable development, is limited. Furthermore, the non-linear empirical models allow for more detailed policy implications. This paper aims to contribute to the question of whether FDI investments can be considered as a mechanism for sustainable development through the perspective of green finance. Therefore, the purpose of this study is to contribute to this gap in the literature and provide a holistic and multidimensional approach to both economic prosperity and environmental protection.

The following part of the paper is structured as follows: the literature review section gives the literature review both from theoretical and empirical backgrounds. Methodology section gives the methodology part with panel data regression analyses. Empirical results section gives the results of the descriptive statistics, the correlation analysis, the results of the cross-sectional dependence, the panel unit root tests, and the panel data analysis, following discussions and the last section concludes the study.

## Literature review

FDI flows have generally targeted countries that offer financial benefits and incentives **s**ince the 1990s [8]. The first study in the empirical literature on FDI and its impact on the environment was conducted by [9]. In this study, the authors examined the environmental impact of North American free trade on 42 countries to evolve the linkage between air quality and economic growth. According to [10], the countries with the lowest environmental standards are the most polluting industries in the world and global pollution increases with trade in these countries. There are several studies in the literature that establish a link between pollution and FDI in both developing and developed countries. While some studies support the pollution hypothesis, others have found no evidence for it. This is due to different econometric methods, different data sources, and alternative conceptual frameworks. Below are some of the literature studies compiled from numerous academic sources.

Multinational companies relocate dirty industries to countries with lax environmental regulations to avoid the costs associated with strict environmental policies, thereby increasing environmental degradation [11]. Some empirical studies document the impact of FDI on environmental degradation, especially from the point of view of the pollution haven effect such as [12] analyzes for 112 countries, [13] for India, [14] for Malaysia, [15] for China, [16] for Group of Twenty (G20) countries, [17] for Turkey, [18] for the top five emitters of greenhouse gas emissions from fuel combustion in developing countries in terms of China, India, Iran, Indonesia and South Africa covering the period of 1982 and 2016, [19] for the 21 developed and developing countries with high carbon emissions between the years 1990 and 2016, [20] for the 50 countries with the highest CO2 pollution in 2010, [21] for Vietnam, [22] for Pakistan. These studies show that foreign direct investment leads to higher consumption of natural resources such as fossil fuels, which in turn results in higher environmental pollution. Additionally, labor and environmentally intensive industries create a pollution haven effect [23]

The pollution halo effect is based on the idea that FDI can bring environmentally friendly technologies and standards to host countries and thereby reduce carbon emissions [24]. Foreign investments, such as technology transfer and the spillover effect of management skills, contribute to improving environmental sustainability in host countries and play an effective role in reducing pollution by increasing energy efficiency in developing countries [25]. FDI enables the export of greener technologies and standards. Through green technologies, these investments lead to a rapid improvement in energy efficiency and a reduction in CO_2_ emissions. It is therefore argued that FDI can improve environmental quality [25]. the halo effect of pollution is supported by [26] for developing countries, [27] for BRIC countries, [28] for Türkiye and [29] for 16 European countries.

Some of the empirical studies show that the "pollution haven" and "pollution halo" hypotheses are simultaneously valid. [30] analyzed the impact of FDI on carbon emissions for 164 countries between the years 1961 and 2004. The analysis of panel data shows that relaxed environmental regulations can increase FDI, but that foreign firms use less polluting technologies than local firms in low-income countries. Furthermore, [31] examine the impact of FDI on environmental quality for 102 countries between the years 2000 and 2015. The results show that the "pollution haven" hypothesis exists for low-income countries, while the "pollution halo" hypothesis holds for middle- and high-income countries. In addition, [27]for BRIC countries, [32] for 20 high-income countries, and [33] for 96 countries with different time intervals argue that these hypotheses are valid simultaneously.

The ecological footprint index is another indicator used in the literature as a proxy for environmental degradation. From the pollution halo effect perspective, developed countries with strict environmental regulations have advanced technologies supporting environmentally friendly production and can transfer these sustainable practices to developing countries leading to a reduction in ecological footprint [34]. [32] examined data on FDI and ecological footprint in 20 countries between 1982 and 2013. The result of the study reveals that the effect of FDI on environmental degradation is negative in developed countries, while it is positive in developing countries. In addition, [35] examined the impact of FDI and economic growth on the ecological footprint in 92 countries during the period 2001 and 2016. The empirical results show that FDI increases the ecological footprint. The halo hypothesis of pollution is not valid. Apart from these results, the variables of economic growth and ecological footprint were found to be negatively related. In addition, [29] used ecological footprint, FDI, GDP, renewable energy, and human capital data for 16 European countries between 1990 and 2019. As a result of the study, it was found that the increase in GDP had a statistically significant and positive effect on the ecological footprint. It has been observed that FDI hurts the ecological footprint. [36] examined the impact of FDI on the ecological footprint of 117 countries in the period between 1984 and 2011. Financial services in high-income countries FDI has been shown to reduce the environmental footprint of production. This shows that the halo hypothesis of pollution in high-income countries is valid.

Furthermore, the nexus between economic development and FDI has been examined from various angles. [37] examined the impact of economic globalization and productivity on environmental quality in emerging countries under the pollution halo hypothesis. The panel estimator was applied in the study with data from 11 emerging economies between the years 1975 and 2017. Econometric analysis reveals that the ecological footprint in emerging countries decreases as the degree of economic globalization increases. It was also found that GDP per capita is positively related to the ecological footprint. It was also found that an increase in total factor productivity increases the ecological footprint. The results of the model estimation show that the pollution halo hypothesis is valid in 11 emerging countries. Furthermore, [35] investigated the impact of FDI and economic growth on the ecological footprint in 92 countries during the years 2001 and 2016. The empirical results show that FDI increases the ecological footprint and the halo hypothesis of pollution is not valid. Apart from these results, economic growth and ecological footprint were found to be negatively correlated.

## Methodology

The sample comprises a selection of the 20 largest CO_2_ emitters and is obtained from the Energy Institute Statistical Review of World Energy 2023 report [38]. The countries with the highest carbon emissions were selected, as environmental degradation is more pronounced with an increase in CO_2_ consumption. China, the United States, India, the Russian Federation, Japan, Iran, Indonesia, Saudi Arabia, Germany, South Korea, Canada, Mexico, Turkey, Brazil, South Africa, Australia, the United Kingdom, Vietnam and Italy are the sample countries. The United Arab Emirates was excluded from the sample due to missing variables and to obtain as balanced a sample as possible. There are two main limitations in the study in terms of time intervals and number of countries. Due to difficulties in obtaining data, these limitations may vary depending on the pollution indicators. The fact that the related data for some countries start with the year 1996 is the most important factor in determining the study period.

Carbon dioxide emissions and ecological footprints have been used as an indicators of environmental degradation similar to the work of [35, 36, 39, 40] Karaduman, (2022). The carbon dioxide measures, *LNCO*_*2*_ are determined by the largest CO_2_ emitters for carbon dioxide equivalents from energy, process emissions, methane, and flaring measured in million tones. The data is drawn from the Energy Institute Statistical Review of World Energy 2023. Ecological footprint data, *EF* which is the only metric that calculates the degree of impact humans’ lifestyle has on the environment, is drawn from the Global Footprint Network (Solarin and Al-Mulali, 2018; [29, 35]). The net inflows for FDI, *LNFDI* net inflows of the balance of payment measured in USD, *LNFDISQ* refers to the squared value of the foreign direct investment measured in USD figures. Related data drawn from the World Bank Database were used. Additionally, this paper has used macroeconomic variables as control variables to observe the effect of FDI on environmental degradation. Gross domestic product, *GDP* growth, and inflation rate, *IR* were used as control variables and drawn from the World Bank Database. The inflation rate is one of the most basic macroeconomic indicators that shows the rate of increase in the general price level of goods and services. An increase in inflation causes the purchasing power of the country’s currency to fall and the prices of goods and services to increase at the same time.

To analyze the impact of FDI investments on environmental degradation panel data regression models have been utilized. The first model indicates carbon emissions and the second model determines the ecological footprint as the indicator of environmental degradation.

To test the effect of FDI on environmental degradation, two-panel data regression models were used.

Model(1); for CO2 emissions

LNCO_2_ = *α* + *β*1LNFDI*it* + *β*2LNFDISQ*it* + *β*3GDP*it* + *β*4IR*it* + *εi*

*Model (2)*; *for ecological footprint*

EF = *α* + *β*1LNFDI*it* + *β*2LNFDISQ*it* + *β*3GDP*it* + *β*4IR*it* + *εi*

i = 1,2, 3,. . . .19

t = 1,2, 3,. . . .27

*α* = constant term

*ε* = error terms

*β* = coefficient of variables

## Empirical results

This section presents the results of the descriptive statistics, the correlation analysis, the results of the cross-sectional dependence, the panel unit root tests, and the panel data analysis. The analysis was carried out using the econometric program STATA 18.

### Descriptive statistics

For mitigating the impact of outliers, the winsorizing method has been used. This paper employs the winsorizing method on all variables by substituting extreme values with those corresponding 1st and 99th percentiles to enhance the robustness of panel data analysis and reduce the sensitivity. The natural logarithm of carbon emissions and FDI is expressed in its natural logarithmic form.

Table 1 shows the mean, standard deviation, minimum, maximum statistics, and normality tests for the dependent, explanatory, and control variables during the related years. The balanced final sample consists of 513 country-year observations between the years 1996 and 2022. During the 27 years, the dataset includes a varying number of country-year observations, and due to the nature of the related variables, the country-year data on these variables start with the year 1996. [41] normality test) was utilized.

**Table 1:** Descriptive statistics of related variables.

Variables	Mean	Standard deviation	Skewness	Kurtosis	Minimum	Maximum	Normality test
LNCO_2_	6.5937	0.9716	0.8870	4.0151	4.1715	9.2593	50.67(0.0000)
EF	2.4484	1.4049	0.5855	2.5346	0.3719	5.9435	27.85(0.0000)
LNFDI	24.9214	1.4042	-14.7024	293.3979	1.6094	27.0358	87.79(0.0000)
LNFDISQ	622.7486	40.3430	-6.5089	110.5350	2.5902	730.9381	93.01(0.0000)
GDP	3.2776	3.5725	-0.52339	4.6209	-13.1267	14.2308	11.13(0.0000)
IR	7.3269	12.8096	4.9487	38.7850	-16.5815	143.6397	306.31(0.0000)

Values in the parenthesis refer to the p values of [41] normality test.

*** indicates a 1% level of significance

The significant values of the normality test confirm that all variables follow the non-normal distribution.

### Pearson correlation analysis

Table 2 gives the Pearson correlation coefficients between the explanatory and control variables. The FDI variable is significantly negatively related to IR which means the inflation level significantly impacts the amount of foreign investments which is similar [42, 43].

**Table 2:** Pearson correlation matrix for explanatory and control variables.

	LNFDI	LNFDISQ	GDP	IR
LNFDI	1.0000			
LNFDISQ	0.9999***	1.0000		
GDP	0.0213	0.0216	1.0000	
IR	-0.2762***	-0.2737***	0.0950	1.0000

***p<0.01

**p<0.05

*p<0.10

### Cross-sectional dependence results

Pesaran’s (2007) test for cross-sectional dependence was used to determine the presence of cross-sectional dependence across panels [44]. Table 3 gives the cross-sectional dependence results Fixed Effect (FE) and Random Effect (RE) panel data models were constructed. Model I indicates the results of a panel data analysis in which the effect of FDI on CO_2_ emissions. Additionally, Model II shows the results of panel data analysis, constructing the dependent variable as the ecological footprint and revealing the impact of FDI on the ecological footprint. The results of the F-test with a statistical value of 4.44 in Model I and 5.40 in Model II strongly indicate that the fixed effect is valid for the respective models. The significant p values of the Pesaran CD test fail to accept the null hypothesis of cross-sectional independence at a 1% level of significance for Model I that cross-sectional dependence exists. Additionally, the significant p values of the Pesaran CD test fail to reject the null hypothesis of cross-sectional independence at a 1% level of significance that cross-sectional dependence does not exist for Model II.

**Table 3:** Cross-sectional dependence results.

Tests	Pesaran CD test	p-value
Model I		
FE model	3.988	0.0001
RE model	4.042	0.0001
Model II		
FE model	1.138	0.2551
RE model	1.076	0.2821

### Panel unit root tests

The econometric analysis is sensitive to the degree of stationarity of the series. Non-stationary series can lead to undesirable regression problems [45]. Since cross-sectional dependence was found for Model I, the second generation panel unit root test was performed. The panel unit root test of [46] was constructed for Model I.

Table 4 gives Pesaran’s CADF test results for Model I.

**Table 4:** Pesaran’s CADF test results for Model I.

*level results*						
	t-bar	cv10	cv5	cv1	Z[t-bar]	P-value
LNCO_2_	-2.054	-2.110	-2.200	-2.380	-1.365	0.008
LNFDI	-2.687	-2.110	-2.200	-2.380	-4.210	0.000
LNFDISQ	-2.678	-2.110	-2.200	-2.380	-4.171	0.000
GDP	-2.807	-2.110	-2.200	-2.380	-4749	0.000
IR	-2.459	-2.110	-2.200	-2.380	-3.186	0.001
*first difference results*						
ΔLNCO_2_	-3.349	-2.110	-2.200	-2.380	-7.183	0.000
ΔLNFDI	-4.333	-2.110	-2.200	-2.380	-11.608	0.000
ΔLNFDISQ	-4.328	-2.110	-2.200	-2.380	-11.585	0.000
ΔGDP	-4.503	-2.110	-2.200	-2.380	-12.370	0.000
ΔIR	-4.051	-2.110	-2.200	-2.380	-10.342	0.000

Since no cross-sectional dependence was determined for Model II, the panel unit root test of the first generation was performed. The panel unit root tests of [46] and [47] panel unit root tests were taken into account. Table 5 indicates the lm-Pesaran-Shin and Breitung-Das test results for Model II. These tests confirmed that all variables are stationary in their level form of 1% and 5%. The test statistics and the corresponding p-values indicate that all series are stationary at the 1% and 5% significance level, *I(1*), at the first difference.

**Table 5:** lm-Pesaran-Shin and Breitung-Das unit root tests for Model II.

	Im-Pesaran-Shin test			
	Levels		First difference	
	with trend	without trend	with trend	without trend
EF	-4.1682***	0.8536	-12.7030***	-12.3469***
LNFDI	-6.9220***	-3.9978***	-13.0314***	-12.9844***
LNFDISQ	-6.9324***	-3.9893***	-13.0307***	-12.9845***
GDP	-11.1434***	-10.3829***	-14.7065***	-14.6998***
IR	-6.4074***	-6.2775***	-12.9960***	-12.5222***
	Breitung- Das test			
	Levels		First difference	
	with trend	without trend	with trend	without trend
EF	-1.6837**	4.1354	-11.4581***	-11.0864***
LNFDI	-3.9330**	-1.7466**	-7.9133***	-15.2386***
LNFDISQ	-3.9478***	-1.7706**	-7.8816***	-15.2563***
GDP	-7.0791***	-9.6633***	-8.4508***	-14.8978
IR	1.9447	-3.0092***	-4.9319***	-7.6844***

Values of the Z-t-tilde-bar are reported for the Im-Pesaran-Shin unit-root test.

Values of lambda statistics are reported for the Breitung test.

*** and ** indicate significance at 1% and 5% level respectively

### Panel data analyses results

Panel Data Analyses were performed for Model I and Model II. Table 6 indicates the Fixed Effect (FE) and Random Effect (RE) panel data results for both models.

**Table 6:** FE and RE panel data analysis results for Model I and Model II.

	Model I		Model II		
	FE	RE	FE	RE	
LNFDI	4.9129	4.7276	1.7789		1.7082
	(1.6679)***	(1.6770)***	(2.4074)		(2.4270)
LNFDISQ	-0.0905	-0.0867	-0.0279		-0.0264
	(0.0329)***	(0.0331)***	(0.0475)		(0.0479)
GDP	-0.0044	-0.0043	0.0041		0.0035
	(0.0035)	(0.0036)	(0.0051)		(0.0052)
IR	-0.0032	-0.0033	-0.0047		-0.0048
	(0.0012)	(0.0012)***	(0.0017)***		(0.0017)***
F test–FE	31.52	130.46	17.78		73.27
Wald chi2(5)–RE					
Goodness of fit (R2)	0.4119	0.4117	0.2229		0.2266
Number of observations	513	513	513		513

For determining the superiority of the fixed effects model or random effect model, the Hausman test is constructed [48]. The statistical results of Prob > Chibar2 = 0.0321 were lower than 5 percent, indicating that the fixed-effects models should be preferred for Model I. Additionally, the statistical results of Prob > Chibar2 = 0.0102 were higher than 5 percent, indicating that the fixed-effects models should be preferred.

### Heteroscedasticity and autocorrelation test results

Panel data models can provide biased results in the case of autocorrelation in the errors and heteroscedasticity within the cross-sections. The Modified Wald test for groupwise heteroscedasticity in fixed-effects regression models was applied to both models to test for groupwise heteroscedasticity.

Table 7 shows the results of the heteroscedasticity test. According to the chi2 statistic of the Modified Wald test for group heteroscedasticity in the FE panel data regression model, the null hypothesis of no heteroscedasticity is rejected; this means that heteroscedasticity is present for both models.

**Table 7:** Modified Wald test for groupwise heteroscedasticity results for models.

H0: sigma(i)^2 = sigma^2 for all i	
Model I	
Chi2(19)	2815.44
Prob>chi2	0.0000
Model II	
Chi2(19)	55301.46
Prob>chi2	0.0000

*Autocorrelation tests* are examined using the *Durbin-Watson test* by [49] and the LBI test is conducted which was developed by [50]. Table 8 shows the autocorrelation test results in the fixed effects model for both models indicating the presence of autocorrelation of errors.

**Table 8:** Autocorrelation test results.

H0: sigma(i)^2 = sigma^2 for all i		
Model (1)	F test that all u_i = 0: F(18,471)	11.15
	Prob > F	0.0000
	Modified Bhargava et al. Durbin-Watson	0.2624
	Baltagi-Wu LBI	0.4254
Model (2)	F test that all u_i = 0: F(18,471)	18.57
	Prob>F	0.0000
	Modified Bhargava et al. Durbin-Watson	0.3095
	Baltagi-Wu LBI	0.4357

Acknowledging the presence of autocorrelation and heteroskedasticity, panel data regression models have been performed using the robust estimator of Driscol-Kraay estimator [51]. Table 9 presents the results of the regressions with robust standard errors and Driscoll-Kraay standard errors.

**Table 9:** Results of the regressions with the Driscoll-Kraay standard error model.

	Model I	Model II
	Driscol Kraay’s standard errors	
LNFDI	-14.7879	-2.9029
	(5.8890)***	(5.3144)
LNFDISQ	0.3125	0.078
	(0.1160)**	(0.1036)
GDP	0.0219	-0.1166
	(0.0090)**	(0.0024)***
IR	-0.0028	-0.0091
	(0.0029)	(0.0036)**
F test–FE	206.29	34.33
Goodness of fit (R2)	0.4189	0.2848
Number of observations	513	513

*** and ** indicate significance at 1% and 5% level respectively

## Discussions

Driscoll-Kraay standard error model shows a significant and negative impact of LNFDI on CO_2_ emissions. This negative sign of the LNFDI variable confirms the validation halo hypothesis. Moreover, the studies by [27, 52] find a negative impact of FDI on CO_2_ emissions. This implies that FDI inflow to these countries leads the clean energy technologies and thereby can reduce the environmental degradation in these countries.

On the other hand, [20] found a significantly positive impact of FDI on CO_2_ emissions for 50 countries between the years 2000 and 2015. Additionally, [12] found a positive linkage between foreign investments and CO_2_ emissions for 112 countries between 1971 and 1997. Furthermore, the results are also consistent with those of [13, 18, 53]. The positive impact of foreign investments on environmental degradation in terms of CO_2_ emissions supports the pollution-haven hypothesis. [19] use carbon emission per capita as a proxy for environmental degradation covering 21 developed and developing countries with high carbon emissions and confirmed the pollution haven hypotheses, specifically in developing countries. Moreover, the results reveal that institutional factors indicating the presence of liberal environmental policies in host countries negatively affect CO_2_ emissions. Additionally, Model I also posits a positive and statistically significant linkage between the variable LNFDISQ and CO_2_ emissions. This negative coefficient of the variable LNFDI and a positive coefficient of the variable LNFDISQ imply the prevalence of a U-shaped pattern for CO_2_ emissions. This empirical finding is similar to the past studies [54]. [53] study on high, middle, and low-income countries and they argue an inverted-U-shaped linkage between FDI and CO_2_ emissions in global and middle-income panels.

The ecological footprint represents environmental degradation more accurately than CO_2_ emissions [55]). The results of the Driscoll-Kraay standard error model for Model II show that FDI and the non-linear form of FDI have no significant influence on ecological footprint. [55] posit a U-shaped relationship between FDI and ecological footprint. They reveal that environmental degradation decreases to a certain level with increasing FDI investment and after this level, increasing FDI increases the environmental degradation. Furthermore, [35] support the strong impact of increased FDI activities on environmental degradation in terms of ecological footprint whereas [36] finds a negative impact of FDI on the ecological footprint. The empirical findings also reveal any linkage between IR and carbon emissions but posit a significant and negative impact on the inflation rate and ecological footprint which shows the need for a holistic and multidimensional approach for both economic prosperity and environmental protection. On the other hand, [56] argue that carbon emissions will begin to decline if IR exceeds 4.7% for the US and 7.5% for the European Union between the years 1960 and 2021. Furthermore, the results show that an increase in GDP leads to a deterioration of the environmental situation in terms of carbon emissions, which means that a higher level of income leads to a higher demand for environmental quality. However, the negative correlation between GDP and EF shows that the higher the GDP, the lower the ecological footprint. This could be explained by the fact that improvements in the economic structure tend to reduce the ecological footprint.

## Conclusions

When countries receive FDI, there are often changes in their industrial structures, energy consumption rates, and economic activities. This could have an effect on environmental sustainability positively through the introduction of greener technologies and negatively through increased industrialization and energy consumption. The process of environmental degradation accelerated worldwide with the Industrial Revolution and the developments that subsequently threatened environmental sustainability. Multinational companies tend to invest more in developing countries, where environmental standards are much more flexible than in industrialized countries. As a result of environmental awareness in industrialized countries, dirty industries are locating their facilities in developing countries that require direct foreign capital investment for their growth and development. The direct inflow of foreign capital therefore leads to a deterioration of environmental quality in developing countries over time. This situation leads to a deterioration of environmental quality in developing countries and an improvement of environmental quality in industrialized countries.

Empirical results reveal a U-shaped relationship between FDI and its non-linear component on carbon emissions. This means environmental degradation decreases to a certain level with increasing FDI investment and after this level, increasing FDI increases the environmental degradation in terms of carbon emissions. The results reveal that the green technology-based production activities of foreign firms inİtially tend to decrease environmental degradation. However in the long run, because of the weak environmental regulation, foreign investments decrease the environmental quality. Moreover, empirical findings also reveal that FDI and the non-linear form of FDI have no significant influence on ecological footprint. Hence, the validity of both the pollution haven hypothesis and the pollution halo hypothesis is rejected and the U-shaped linkage between FDI and carbon emissions is confirmed.

Environmental sustainability requires a local, national, and global challenge. Global cooperation and political initiatives are essential to ensure a sustainable future. Compliance with international agreements by low-income countries should be strengthened and multinational companies should be encouraged to invest in these countries with clean energy and technology. The impact of a carbon tax could be included in the models to show the impact of carbon pricing on FDI. The renewable energy sector and associated employment opportunities should be encouraged by increasing taxes on companies operating in the fossil fuel sector. The switch to clean energy sources and the introduction of environmentally friendly technologies should also eliminate the negative effects of foreign investments. Again, by switching to renewable energy sources and ensuring green growth, initiatives should be taken to ensure that economic growth does not lead to environmental degradation but rather contributes to environmental sustainability. Environmentally friendly employment opportunities should be created in environmentally friendly sectors by providing incentives and subsidies to companies that can demonstrate this. Furthermore, developing less carbon-intensive projects should be incentivized. To keep CO_2_ emissions and the ecological footprint at an acceptable level, approval procedures for FDI projects should be well managed. Policymakers are adapting strategies to promote sustainable development while reducing the environmental impact. A holistic and multidimensional approach should be redesigned for both economic prosperity and environmental protection. By promoting international cooperation, strengthening the institutional framework, and encouraging the transfer of clean technologies, the world can create a sustainable environment for future generations by meeting the requirements for the transition to a low-carbon economy.
